# Identification of a Neuropeptide S Responsive Circuitry Shaping Amygdala Activity via the Endopiriform Nucleus

**DOI:** 10.1371/journal.pone.0002695

**Published:** 2008-07-16

**Authors:** Susanne Meis, Jorge Ricardo Bergado-Acosta, Yuchio Yanagawa, Kunihiko Obata, Oliver Stork, Thomas Munsch

**Affiliations:** 1 Institut für Physiologie, Otto-von-Guericke-Universität, Magdeburg, Germany; 2 Abteilung für Molekulare Neurobiologie, Institut für Biologie, Otto-von-Guericke-Universität, Magdeburg, Germany; 3 Department of Genetic and Behavioral Neuroscience, Gunma University Graduate School of Medicine, Maebashi, Japan; 4 Neuronal Circuit Mechanisms Research Group, Obata Research Unit, RIKEN Brain Science Institute, Wako, Japan; Ludwig Maximilians University Munich, Germany

## Abstract

Neuropeptide S (NPS) and its receptor are thought to define a set of specific brain circuits involved in fear and anxiety. Here we provide evidence for a novel, NPS-responsive circuit that shapes neural activity in the mouse basolateral amygdala (BLA) via the endopiriform nucleus (EPN). Using slice preparations, we demonstrate that NPS directly activates an inward current in 20% of EPN neurons and evokes an increase of glutamatergic excitation in this nucleus. Excitation of the EPN is responsible for a modulation of BLA activity through NPS, characterized by a general increase of GABAergic inhibition and enhancement of spike activity in a subset of BLA projection neurons. Finally, local injection of NPS to the EPN interferes with the expression of contextual, but not auditory cued fear memory. Together, these data suggest the existence of a specific NPS-responsive circuitry between EPN and BLA, likely involved in contextual aspects of fear memory.

## Introduction

The 20 residue peptide NPS, termed after its N-terminal serine residue, has recently been identified as the endogenous agonist for the orphan G-protein coupled GPR154 receptor now referred to as NPSR. Analysis in heterologous expression systems revealed a likely G_q_ protein-mediated mobilization of intracellular Ca^2+^, suggesting that NPS may enhance neuronal excitability [1]. Moreover, amino acid residues critical for the NPS activity in cultured cells and *in vivo* have been identified [2]. However, cellular effects have not been described in native neurons so far.

In the brain, NPS is expressed in three brainstem nuclei (locus coeruleus, lateral parabrachial nucleus and principle trigeminal nucleus) as well as a few scattered neurons in the medial amygdala and dorsomedial hypothalamus. Likewise, the NPS receptor shows a distinct expression pattern across various cortical and subcortical regions, hence indicating the existence of a highly specialized NPS circuitry. Based on expression pattern and NPS application *in vivo*, roles in olfaction, anxiety, arousal, hippocampus-dependent learning and memory, as well as energy balance and food intake have been suggested [3]–[6].

Anxiety, arousal and hippocampus-dependent memory formation depend critically on the function of the basolateral amygdala (BLA). For example, the BLA is characterized by a high number of benzodiazepine receptors and mediates the GABAergic control of anxiety [7]. The BLA also supports mnemotic functions as it is critically involved in stress modulation of hippocampus-dependent memory and serves in turn as entry site for hippocampal information to the amygdala during, e.g., contextual fear conditioning for review, see [8], [9]. Thus we hypothesized that the BLA may be involved in NPS functions related to anxiety and memory formation. However, although NPSR is highly expressed in the neighboring areas such as medial and cortical amygdala, and particularly in the endopiriform nucleus (EPN), the BLA itself shows only trace amounts of NPSR mRNA [6].

In the current study we investigated the role of NPS in the control of neuronal activity in the EPN and its potential to modulate activity in the BLA as well as BLA-dependent expression of conditioned fear behavior. We applied electrophysiological and pharmacological techniques in the slice preparation of the mouse amygdala *in vitro* to characterize, for the first time, direct and indirect cellular effects of NPS in native neuronal cells. Our data reveal a hitherto undescribed neuronal circuitry shaping activity patterns in the BLA via the EPN. With local administration of NPS to the EPN *in vivo*, we furthermore gained evidence for an involvement of this circuitry in the retrieval of contextual aspects of fear memory.

## Results

### NPS stimulates GABA-mediated synaptic activity in BLA projection neurons

**Figure 1:**
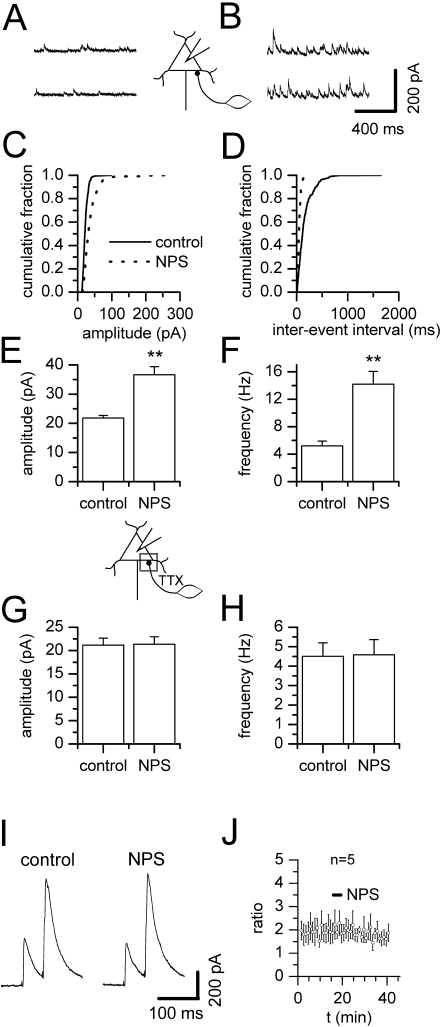
NPS stimulates GABAergic input of BLA projection neurons. In BLA projection neurons, NPS stimulates GABA-mediated synaptic activity dependent on action potential propagation. Examples of GABAergic sIPSCs recorded in a BLA projection neuron before (A) and during action of NPS (B). (C) Cumulative amplitude and (D) inter-event interval histograms obtained from the same neuron shown in (A, B) before addition of NPS and after a steady-state effect had been reached. (E) sIPSC amplitude and (F) frequency pooled during control conditions and after addition of NPS demonstrates a significant increase in sIPSC amplitude as well as frequency. (G) mIPSCs recorded in the presence of TTX were unchanged in amplitude as well as (H) frequency by addition of NPS. (I) Representative traces of IPSCs recorded before and after addition of 200 nM NPS. (J) Time course of mean paired-pulse ratio of eIPSCs showing lack of NPS effects on paired-pulse facilitation in BLA projection neurons. **, p<0.01. Bar, time of NPS addition.

Holding recorded projection neurons at 0 mV for extended periods of time did not lead to changes in activity of GABA_A_ mediated sIPSCs in the absence of NPS. Application of 200 nM NPS with the bathing solution, however, strongly increased activity of sIPSCs in 12 out of 12 cells tested (Figure 1). Typical current traces under control conditions (Figure 1A) and during the presence of NPS (Figure 1B), as well as cumulative amplitude (Figure 1C) and inter-event interval (Figure 1D) histograms illustrate the raise in amplitude as well as the shortening of inter-event intervals upon addition of NPS in a representative neuron. Mean values for amplitude and frequency are shown in Figure 1E and F, respectively. The average maximal amplitude of sIPSCs changed significantly from 21.8±0.8 pA in the absence to 36.6±2.8 pA in the presence of NPS (Figure 1E, n = 12, p = 0.0005), paralleled by a significant increase in average frequency from 5.2±0.7 Hz to 14.2±1.9 Hz (Figure 1F, n = 12, p = 0.0005). In order to rule out that holding neurons at 0 mV for the duration of the experiment leads to synaptic plasticity at the inputs to the recorded cells, we performed a series of experiments with a high concentration of BAPTA (10 mM) in the pipette solution. Also, under these experimental conditions the average maximal amplitude of sIPSCs changed significantly from 14.1±0.9 pA in the absence to 35.8±3.5 pA in the presence of NPS (n = 4, p = 0.0036, data not shown), accompanied by a significant increase in average frequency from 4.2±0.8 Hz to 14.7±1.9 Hz (n = 4, p = 0.0036, data not shown).

Stimulation of GABA-mediated sIPSCs in projection neurons may result from activation of NPS receptors located at GABAergic presynaptic terminals, leading to an increase in intracellular Ca^2+^ as specific for cellular NPS action [1] and thereby to an augmented GABA release. We tested this possibility by analyzing miniature IPSCs (mIPSCs) in the presence of TTX to block action potential dependent transmitter release. Under these experimental conditions, neither amplitude (Figure 1G) nor frequency (Figure 1H) were altered by NPS. Mean amplitude amounted to 21.2 pA±1.5 pA before and 21.3±1.6 pA (n = 7) after addition of the peptide, while frequency averaged to 4.5±0.7 Hz and 4.6±0.8 Hz (n = 7), respectively. Neither values were significantly different (p = 0.4375, p = 0.9375). Thus, the increase in GABA release as seen as an increase in sIPSCs seems to rely on action potential propagation and may result either from direct or indirect excitation of interneurons. To further rule out a direct effect of NPS on presynaptic calcium influx we examined paired pulse facilitation of evoked IPSCs (eIPSCs) in BLA projection neurons. In all cells tested, amplitudes of eIPSCs were unaffected by application of 200 nM NPS (Figure 1I). The amplitude of the second IPSC with respect to the amplitude of the first IPSC was 185.9±36.9% (n = 5) (Figure 1I). A change in paired-pulse facilitation ratio after addition of NPS was not observed (Figure 1J).

### NPS effects on BLA interneurons

**Figure 2:**
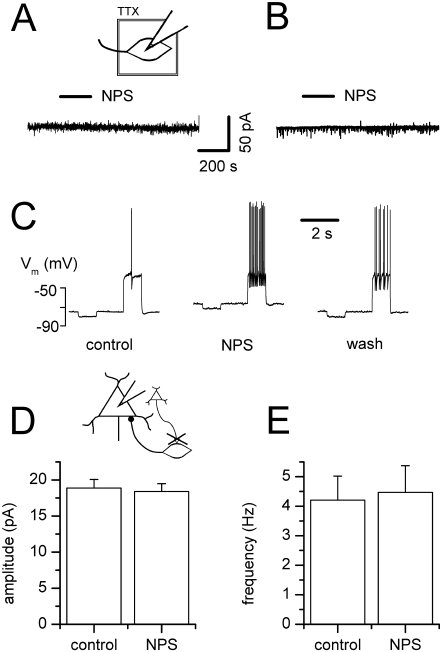
NPS effects in BLA interneurons. Excitatory synaptic transmission is indispensable for the NPS action. (A) In the continuous presence of TTX to block synaptic transmission, application of NPS (as indicated by bar) produces no effect on holding current and thus shows no direct postsynaptic induction of an inward current in BLA interneurons nor (B) intercalated cells. (C) Under current-clamp conditions, NPS application induces a depolarizing response associated with increased spike activity triggered upon depolarizing current injections in BLA interneurons. (D, E) Block of excitatory synaptic transmission by NBQX and AP5 abolishes the effect of NPS on (D) sIPSCs amplitude as well as (E) frequency recorded from BLA projection neurons.

NPS did not exert direct postsynaptic effects on recorded local interneurons in the presence of TTX, indicated by a lack of effect on membrane holding current (Figure 2A, n = 8). This also applied to intercalated neurons (Figure 2B, n = 4), which were recently shown to mediate feedforward inhibition into the basolateral nucleus [10].

By contrast, in BLA interneurons, NPS induced a membrane depolarization from resting membrane potential (−77.4±1.8 mV, n = 10) under current-clamp conditions, with an average maximal amplitude of 5.4±0.9 mV (n = 10). Typical membrane potential responses to the current protocol composed of alternating negative (−25 pA, 1 s) and positive (+175 pA, 1 s) current pulses are shown in Figure 2C. NPS action was accompanied by an increase in presumably glutamatergic depolarizing synaptic events. The input membrane resistance remained unaltered at 480.6±78.2 MΩ before and 501±84.8 MΩ during NPS action (n = 10, p = 0.2754), or 437.3±106.4 MΩ when membrane potential was manually clamped at resting potential at the peak of the NPS effect (n = 6, p = 0.4375), respectively. Mean spike frequency elicited by positive current injections adjusted around spike threshold generation (+25 to +175 pA) increased significantly from 1.6±0.7 Hz (n = 10) before addition of NPS to 10.1±1.4 Hz (n = 10, p = 0.002) during maximal drug action, but persisted unchanged whilst manual clamp conditions (1.7±1.0 Hz, n = 6, p = 0.3750, data not shown).

As interneurons were excited but showed no direct postsynaptic current response upon NPS application, excitatory synaptic transmission is most likely prerequisite for the increase in GABA release seen in projection neurons. Indeed, blocking excitatory synaptic transmission by application of NBQX and AP5 abolished the effect on sIPSCs seen in projection neurons (Figure 2D, E). Mean control amplitudes of 18.9±1.2 pA (n = 8) resembled values in the presence of NPS of 18.4±1.1 pA (n = 8, p = 0.2500). Likewise, frequency averaged to 4.2±0.8 Hz before and 4.5±0.9 Hz after addition of NPS (n = 8, p = 0.3125).

### NPS stimulates glutamatergic synaptic activity in BLA interneurons

**Figure 3:**
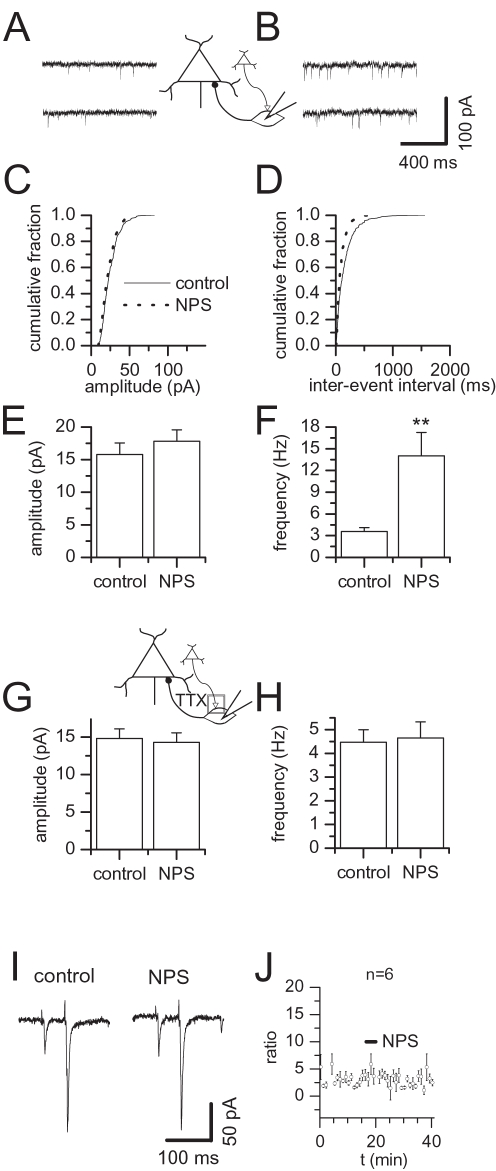
NPS stimulates glutamatergic input of BLA interneurons. NPS stimulates glutamatergic synaptic activity dependent on action potential propagation in BLA interneurons. (A) Examples of glutamatergic sEPSCs recorded in a BLA interneuron before and (B) during action of NPS. (C) Cumulative amplitude and (D) inter-event interval histograms obtained from the same neuron shown in (A, B) before addition of NPS and after a steady-state effect had been reached. (E) sEPSC amplitude and (F) frequency pooled during control conditions and after addition of NPS demonstrates a significant increase in sEPSC frequency. (G) mEPSCs recorded in the presence of TTX were unchanged in amplitude as well as (H) frequency by addition of NPS. (I) Representative traces of EPSCs recorded before and after addition of 200 nM NPS. (J) Time course of mean paired-pulse ratio of eEPSCs showing lack of NPS effects on paired-pulse facilitation in BLA interneurons. **, p<0.01. Bar, time of NPS addition.

Direct evidence for an increase of excitatory input into local interneurons was achieved by recording glutamatergic synaptic activity in interneurons. The modulation of sEPSCs by NPS is exemplified as current traces before (Figure 3A) and after (Figure 3B) application of the drug and summarized as cumulative amplitude (Figure 3C) and inter-event interval (Figure 3D) histograms for a representative neuron. Mean amplitudes were not changed significantly and amounted to 15.8±1.7 pA in the absence and 17.8±1.7 pA in the presence of NPS (Figure 3E, n = 8, p = 0.1953), compared to a significant shift in average frequency changing from 3.6±0.5 Hz to 14.0±3.2 Hz (Figure 3F, n = 8, p = 0.0078). This effect was not mediated by a mechanism on-site presynaptic terminals, as it was eliminated by block of action potentials (Figure 3G, H). In the presence of TTX, amplitude as well as frequency of mEPSCs under control and NPS condition were alike, amounting to 14.8±1.3 pA and 14.3±1.3 pA (Figure 3G, n = 6, p = 0.4375) or 4.5±0.5 Hz and 4.7±0.7 Hz, respectively (Figure 3H, n = 6, p = 0.5625). Also, a direct presynaptic effect of NPS was examined by paired pulse stimulation of EPSCs in BLA interneurons in the presence of 10 µM bicuculline methiodide. In all cells tested, amplitudes of evoked EPSCs (eEPSCs) were unaffected by application of 200 nM NPS (Figure 3I). The amplitude of the second EPSC with respect to the amplitude of the first EPSC was 337.9±30.6% (n = 6) (Figure 3I). Again, a significant change in paired-pulse ratio after addition of NPS was not observed (Figure 3J).

### NPS stimulates glutamatergic synaptic activity in BLA projection neurons

**Figure 4:**
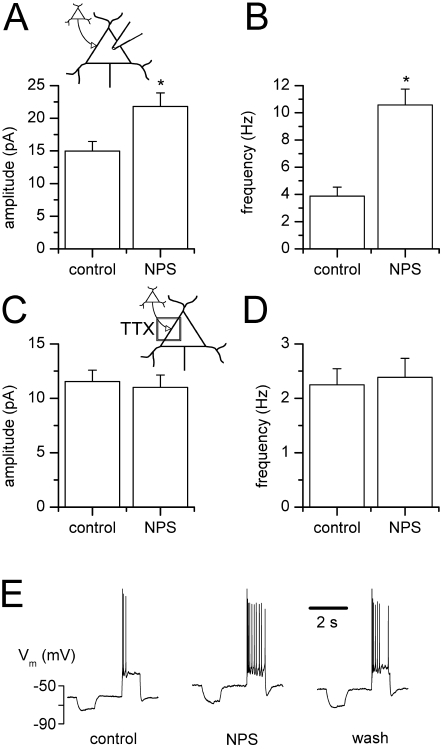
NPS stimulates glutamatergic input of BLA projection neurons. NPS stimulates glutamatergic synaptic activity dependent on action potential propagation in BLA projection neurons. (A) sEPSC amplitude and (B) frequency pooled during control conditions and after addition of NPS demonstrates a significant increase in sEPSC amplitude as well as frequency. (C) mEPSCs recorded in the presence of TTX were unchanged in amplitude as well as (D) frequency by addition of NPS. (E) Under current-clamp conditions, NPS application induces a depolarizing response associated with increased spike activity triggered upon depolarizing current injections in a subset of BLA projection neurons. *, p<0.05.

Excitation may arise from BLA projection neurons, which were shown to be subject to feedforward and feedback inhibition. Activation of projection neurons could thus evoke excitation of local interneurons providing feedback GABAergic inputs. Nevertheless, projection neurons displayed no shift in membrane holding current in response to NPS application in the presence of TTX, thereby showing no direct effect of the peptide on postsynaptic conductance (data not shown, n = 6). On the other hand, average amplitude and frequency of sEPSCs in projection neurons were augmented (Figure 4A, B). In 6 out of 7 projection neurons, amplitude increased significantly from a control value of 15.0±1.5 pA to 21.8±2.1 pA in the presence of NPS (Figure 4A, n = 6, p = 0.0313), while frequency rose simultaneously from 3.9±0.7 Hz to 10.6±1.2 Hz (Figure 4B; n = 6, p = 0.0313). This effect was dependent on spike propagation, as it was abolished in the presence of TTX. Mean amplitude of mEPSCs amounted to 11.5±1.0 pA before and 11.0±1.1 pA after addition of NPS (Figure 4C, n = 7, p = 0.1094), while frequencies averaged 2.3±0.3 Hz under control condition and 2.4±0.4 Hz in the presence of the peptide (Figure 4D, n = 7, p = 0.1563).

This excitation did not lead to a depolarization under current clamp condition in 12 out of 20 neurons. In the remaining 8 cells, NPS evoked a membrane depolarization averaging to 10.6±2.6 mV (n = 8) from the resting membrane potential of −75.5±0.9 mV (n = 8). A representative example is shown in Figure 4E. The input membrane resistance remained unchanged at 447.5±51.9 MΩ before and 570±84.3 MΩ after addition of NPS (n = 8, p = 0.1484), or 445.3±54.8 MΩ when membrane potential was manually clamped back to resting potential at the peak of the NPS effect (n = 3, p = 1.25), respectively. Mean spike frequency elicited by positive current injections adjusted around spike threshold generation (+25 to +150 pA) increased significantly from 3.9±1.2 Hz (n = 8) before addition of NPS to 9.5±1.2 Hz (n = 8, p = 0.0078) during maximal drug action, but persisted unchanged whilst manual clamp conditions (2.8±1.2 Hz, n = 3, p = 0.5, data not shown).

As projection neurons are not directly activated by NPS, and mEPSCs are not increased by NPS application, the excitation mediated by NPS finally leading to an enhancement of sIPSCs in BLA projection neurons has to develop outside the basolateral amygdala.

### NPS effects on neurons of the endopiriform nucleus

**Figure 5:**
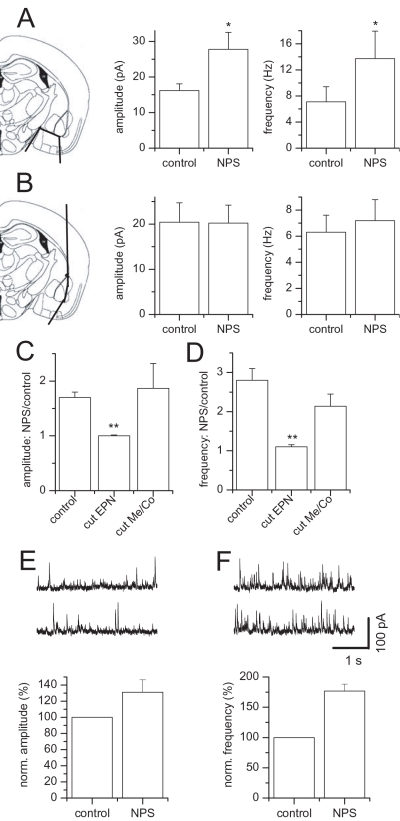
Effects of mechanical circuit dissection. (A) Cuts separating medial/cortical (Me/Co) and BLA do not interfere with the significant increase in amplitude as well as frequency of sIPSCs recorded from BLA projection neurons after addition of NPS. (B) Cuts detaching endopiriform nucleus from BLA eliminate NPS action upon sIPSCs in BLA projection neurons. (C, D) Only dissection in between EPN and BLA diminish (C) amplitude or (D) frequency ratio: NPS/control significantly. (E, F) Examples of GABAergic sIPSCs recorded in a BLA projection neuron before (E, top traces) and after puff application of NPS (F, top traces) into the ventral EPN. Mean normalized amplitude (E) and frequency (F) of sIPSCs during control conditions and after puff application of NPS. *, p<0.05; **, p<0.01. Illustrations modified from [37].

In coronal slices on level with the amygdala, receptor mRNA was clearly expressed in the medial/cortical amygdala and endopiriform nucleus [1]. We therefore tested NPS stimulation of sIPSCs in BLA projection neurons in slices differently dissected. Recordings from isolated BLA slices revealed the absence of significant NPS induced changes in sIPSC activity. Mean amplitudes amounted to 22.1±3.1 pA before and 22.4±3.6 pA after NPS application (p = 0.8467), while frequency was 10.8±2.5 Hz and 10.2±2.1 Hz (n = 11, p = 0.7027, data not shown), respectively. Cuts aimed to divide the BLA from the medial/cortical amygdala did not prevent the effect of NPS on sIPSCs in BLA projection neurons (Figure 5A). Upon addition of NPS, amplitudes changed significantly from 16.2±1.9 pA to 27.8±4.8 pA (Figure 5A, n = 6, p = 0.0313) paralleled by a significant increase in frequency from 7.1±2.3 Hz to 13.7±4.2 Hz (Figure 5A, n = 6, p = 0.0313). In contrast, disconnection of BLA and EPN diminished the effect induced by NPS significantly (Figure 5B). Amplitudes amounted to 20.4±4.3 pA before and 20.2±4.0 pA after NPS application (p = 0.5625), while frequency was 6.3±1.3 Hz and 7.2±1.6 Hz (Figure 5B, n = 6, p = 0.0625), respectively. Under control conditions, the amplitude ratio: NPS to control was 1.7±0.1 in intact slices (n = 12) compared to 1.0±0.02 in slices with BLA disconnected to EPN (n = 6, p = 0.0009) or 1.9±0.5 with cuts separating BLA from medial/cortical amygdala (Me/Co, Fig. 5C, n = 6, p = 0.4260), respectively. The frequency ratio: NPS to control was 2.8±0.3 in intact slices, 1.1±0.06 in slices with cuts detaching BLA from EPN (p = 0.0009) and 2.1±0.3 with dissection in between BLA and medial/cortical amygdala (Figure 5D, n = 6, p = 0.1223).

Cutting along the external capsule might not only disconnect the EPN from the BLA, but could also destroy fibers running within the capsule originating in other cortical areas. We therefore examined whether puffing NPS locally into the EPN could induce an increase in sIPSC activity in BLA projection neurons. Local puffs of NPS for 10–20 s into the ventral EPN increased activity of sIPSCs in BLA projection neurons in 5 out of 11 slices tested (Figure 5E, F). Typical current traces under control conditions (Figure 5E) and after local NPS puffs (Figure 5F) illustrate the rise in amplitude as well as frequency of sIPSCs upon local puff application of NPS in a representative projection neuron. The normalized mean amplitude was increased to 131.2±15.3% (n = 5) compared with control values (Figure 5E) and the normalized mean frequency raised to 176.8±11.5% (n = 5) of control values (Figure 5F) upon puff application of NPS. In contrast, local NPS puffs into the medial/cortical amygdala did not lead to significant (p>0.05) changes in either the frequency or the amplitude of sIPSCs recorded from BLA projection neurons (normalized mean frequency: 93.9±9.8%; normalized mean amplitude: 97.8±5.0%, n = 8).

**Figure 6:**
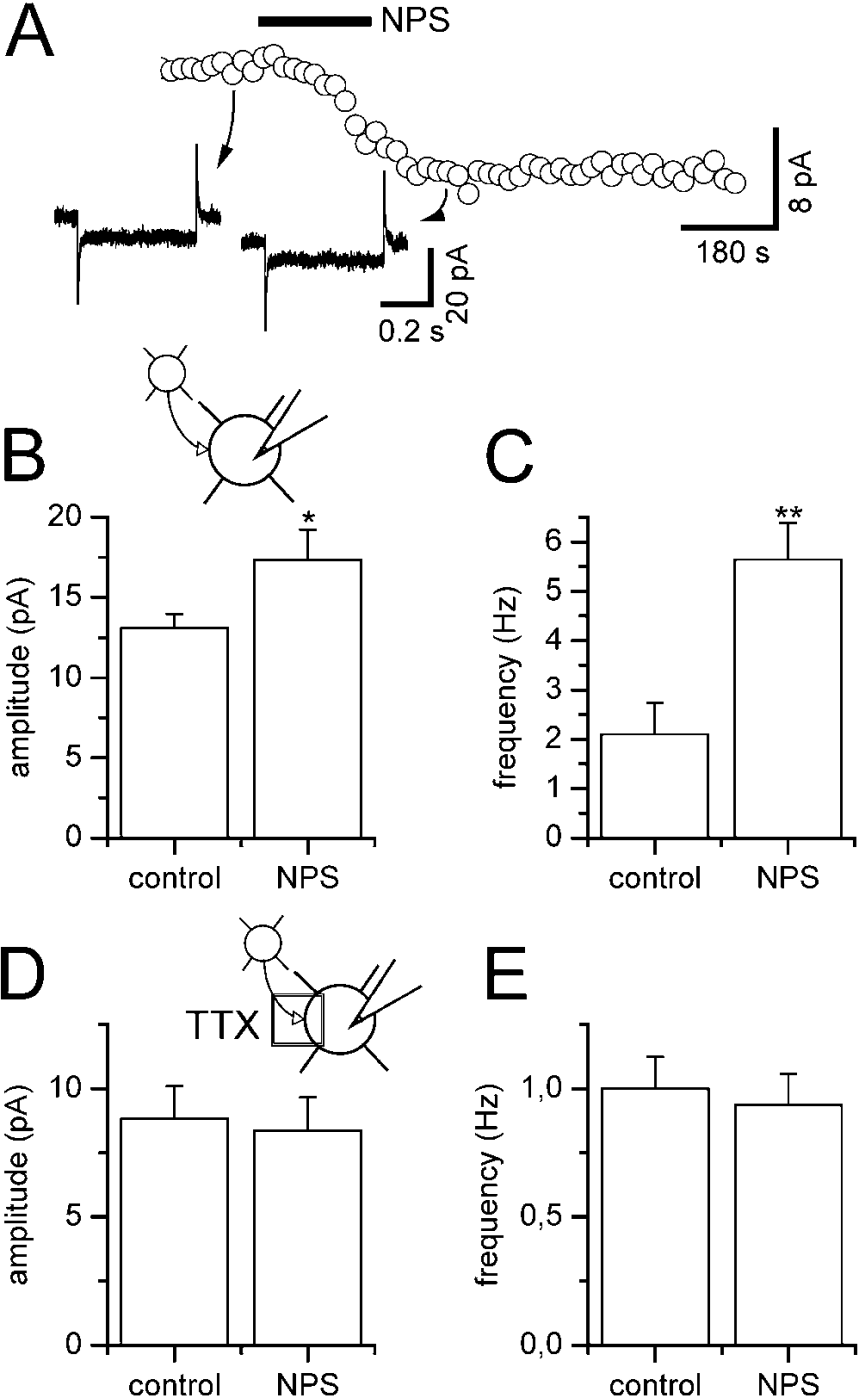
NPS effects on EPN projection neurons. NPS effects on neurons of the endopiriform nucleus. (A) In the presence of TTX, NPS evokes a transient inward current from a holding potential of −70 mV associated with an increase in input resistance as indicated by responses to small hyperpolarizing test pulses. (B) sEPSC amplitude and (C) frequency pooled during control conditions and after addition of NPS demonstrates a significant increase in sEPSC amplitude as well as frequency. (D) mEPSCs recorded in the presence of TTX were unchanged in amplitude as well as (E) frequency by addition of NPS. *, p<0.05; **, p<0.01.

Application of NPS elicited an inward current in 11 out of 49 neurons encountered in the endopiriform nucleus in the presence of TTX (Figure 6A). Mean maximal amplitude amounted to 20.0±3.2 pA (n = 11) at the holding potential of −70 mV. The inward current was associated with a slight albeit significant increase in membrane resistance as assessed by voltage steps (Figure 6A, 500 ms, −10 mV) amounting to 626.8±79.3 MΩ before and 714.8±90.9 MΩ after addition of NPS (n = 11, p = 0.0049). This may be due to an inhibition of K^+^ currents or activation of a voltage-dependent cationic conductance [11].

In addition to these direct postsynaptic effects in a subset of neurons, glutamatergic synaptic activity was strongly increased. In 8 out of 9 cells, mean values for sEPSC amplitude changed significantly from 13.1±0.9 pA in the absence to 17.3±1.9 pA in the presence of NPS (Figure 6B, n = 8, p = 0.0234), accompanied by a significant increase in average frequency from 2.1±0.6 Hz to 5.6±0.7 Hz (Figure 6C, n = 8, p = 0.0078). These effects were inhibited by addition of TTX, leading to amplitudes of 8.8±1.3 pA before and 8.4±1.3 pA after addition of NPS (Figure 6D, n = 5, p = 0.1250) and frequencies of 1.0±0.1 Hz and 0.9±0.1 Hz (n = 5, p = 0.1250), respectively (Figure 6E).

### Behavioral NPS effects

**Figure 7:**
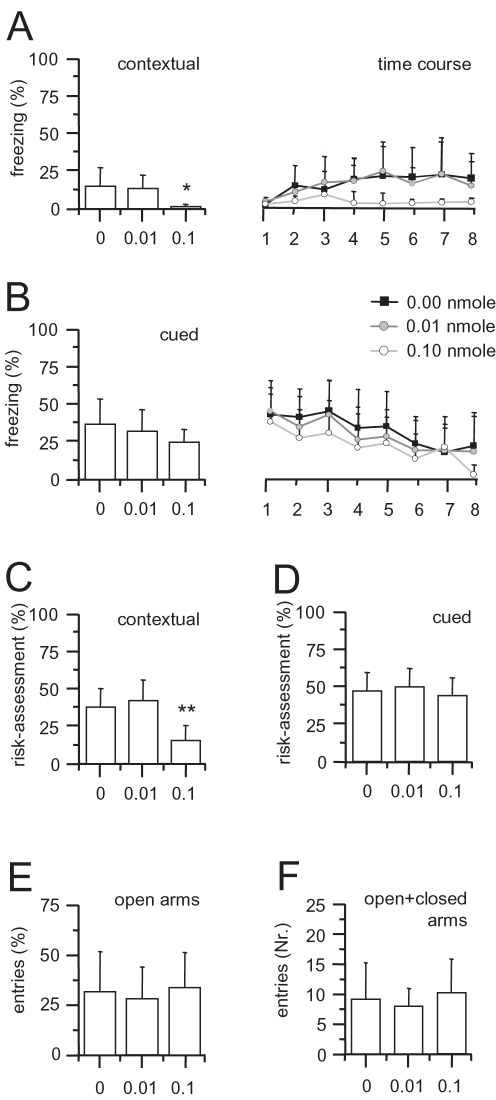
Behavioral NPS effects. A selective reduction of contextual fear responding was observed upon NPS administration. (A) Injection of 0.1 nmole NPS to the endopiriform nucleus (n = 14), resulted in significantly reduced freezing behavior during contextual fear memory retrieval, as compared to vehicle injected controls (n = 12). At a dose of 0.01 nmole, NPS had no such effect (n = 12). Time course analysis of conditioned freezing behavior revealed a continuous reduction during the contextual retrieval session. For better comparison the 2-min context exposure was dissected into eigth intervals of either 10 s 1,3,5,7) or 20 s (2,4,6,8) length, analogous to those in cued retrieval. (B) In cued fear memory retrieval, a somewhat reduced freezing response was observed in the 0.1 nmole group, but this changes failed to reach significance level. The reduction was mostly observed during inter-stimulus-intervals and hence may have resulted from the animals' reduced fear response to the background context, whereas the responses during tone exposure were almost identical between groups. 1,3,5,7, conditioned stimluli (CS+, 10 s), 2,4,6,8 inter-stimulus-intervals (20 s). (C, D) A similar specific reduction during contextual versus cued memory retrieval was observed for a second defensive behavior, risk-assessment. (E, F) No change of anxiety-related behavior was apparent in an elevated plus maze. All values are means±SEM; *, p<0.05; compared to vehicle, **, p<0.01.

Injection of NPS to the EPN resulted in a reduction of contextually conditioned fear behavior without apparent deficits in auditory cued fear memory or in general anxiety level (Fig. 7).

In detail, during the retrieval of contextual fear memory, NPS treatment had a significant effect on the expression of typical rodent defensive behaviors, i.e. freezing (F_2,37_ = 4.679, p = 0.016) and risk assessment (F_2,37_ = 11.084, p = 0.000). Planed comparison revealed a reduction of freezing (1.45±2.51% compared to 15.09±16.32% for vehicle treatment, p = 0.009) that was largely continuous throughout the contextual retrieval session (Fig. 7A), as well as risk assessment behavior (15.35±11.81% compared to 37.48±15.91% in the vehicle group, p = 0.001; Fig 7C) in the 0.1 nmole NPS treatment group. Animals treated with the lower dose of 0.01 nmole NPS failed to show significant change from vehicle treated controls (freezing: 13.28±14.86%; risk assessment: 41.75±18.57%). NPS treatment at either dose failed to significantly affect freezing (F_2.37_ = 1.60, p = 0.216) or risk assessment behavior (F_2.37_ = 0.425, p = 0.657) during auditory cued fear memory retrieval, although a trend for somewhat lower freezing values was observed in both NPS treatment groups (24.52±12.51% at 0.1 nmole and 31.91±19.31% at 0.01 nmole, compared to 36.46±19.66% for vehicle controls; Fig. 7B, D). Temporal analysis revealed that the somewhat reduced freezing rate was apparent mostly during inter-stimulus-intervals in the 0.1 nmole NPS group, however, significance level was not reached at any point. Finally, in order to address anxiety-related effects on fear memory performance, we determined generalization to a neutral tone (CS−). Again, we observed no significant effect of treatment on freezing behavior (F_2.37_ = 1.659, p = 0.205), although the response was somewhat reduced in the 0.1 nmole group (7.05±10.57%, compared to 12.30±10.94% in the 0.01 nmole group and 18.01±16.17% in vehicle controls). Similarly, no effect was observed on risk-assessment behavior (F_2.37_ = 2.071, p = 0.141, 0.1 nM 36.79±14.66% in the 0.1 nmole group, 48.28±16.95% in the 0.01 nmole group and 37.46±15.78% in vehicle controls).

To further address potential anxiety-related effects of NPS, an elevated plus maze analysis was introduced to our experiments, immediately prior to the fear memory retrieval. Here, no significant effect of NPS treatment was observed on anxiety-like behavior or exploratory activity, as time on open arms (F_2.37_ = 0.176, p = 0.839), the % entries to open arms (F_2.37_ = 0.297, p = 0.744), and total number of arm entries (F_2.37_ = 0.471, p = 0.627) remained unchanged (Fig. 7E, F).

## Discussion

In the current study we identify a previously unknown, NPS-responsive circuitry that controls neural activity in the BLA via the endopiriform nucleus. We demonstrate that NPS exerts direct actions on cells in the EPN and enhances glutamatergic synaptic transmission in the great majority of encountered neurons. Cut experiments reveal that input from the EPN is required for NPS modulation of GABA_A_-mediated feedback and feedforward inhibition in the BLA. This circuitry may be functional in the context of conditioned fear expression, as local application of NPS to the endopiriform nucleus reduces the expression of contextual, but not cued fear memory in mice.

Our data demonstrate that while projection neurons in the BLA receive increased excitatory input upon NPS stimulation, only a subpopulation of those neurons are driven to generate action potentials. At the same time, GABA_A_ receptor dependent inhibitory transmission is enhanced, likely through both, feedback inhibition via recurrent excitatory connections [12] and feedforward inhibition, as indicated by a former study showing a multiphasic IPSP following the initial depolarizing potential upon stimulation of the EPN [13].

Thus NPS appears to be able to selectively shape information processing in BLA circuits. However, neither direct postsynaptic actions of NPS on interneurons and projection neurons are encountered in the BLA nor effects on miniature postsynaptic currents independent of action potential propagation. Still, we cannot exclude that NPS may directly modulate voltage-dependent ionic conductances in the BLA via second messenger cascades not investigated in the present study. Also, analysis of mIPSCs may underestimate mechanisms of inhibition upstream of Ca^2+^ entry, namely inhibition of Ca^2+^ channels or activation of K^+^ channels within the nerve terminal. However, our experiments revealed that NPS neither affects paired-pulse facilitation of IPSCs in BLA projection neurons nor paired-pulse facilitation of EPSCs in interneurons, hence arguing against a modulation of presynaptic mechanisms through NPS receptors localized on the relevant terminals. In summary, these data suggest that the observed response in BLA neurons originated from another structure that is endowed with the NPS receptor and preserved connections to the BLA in the frontal slice preparation.

Several lines of evidence suggest the endopiriform nucleus as the source of this NPS dependent modulation: the NPS receptor is expressed most abundantly in this region [6], direct projections from the piriform cortex and endopiriform nucleus to the BLA have been described [14]–[16], and stimulation of the ventral endopiriform nucleus can evoke excitatory postsynaptic potentials in the BLA [13]. Indeed, our data suggest that the observed modulation of neural activity in the BLA through NPS may be mediated by the EPN. Firstly, a subpopulation of neurons of the EPN developed an inward current upon application of NPS in the presence of TTX. Secondly, most neurons in the EPN were subject to elevated excitatory synaptic transmission, presumably due to the prominent intrinsic excitatory connections. Thirdly, disconnection of BLA and EPN prevented the NPS-induced increase of sIPSCs in BLA projection neurons. Fourthly, recordings from isolated BLA slices showed a complete absence of NPS-effects on sIPSC activity. And fifthly, puffing NPS locally into the EPN caused an increase in sIPSC activity comparable to that observed after bath application of NPS.

The EPN is regarded as part of the olfactory cortex, and closely associated with the piriform cortex (PC). In fact, NPS receptors are also expressed in lower layers of the PC, indicating that they may form a functional NPS-responsive unit with the EPN/PC area. The previously observed involvement of the EPN in the integration of olfactory and gustatory information has led to the suggestion that it may via its efferent connections to the amygdala be involved in food selection and – more generally – in emotional reactions related to chemical sensation [17]. Moreover, the EPN is well known for its role in epileptogenic properties, and kindling-induced epileptiform potentials in PC slices were shown to originate in the EPN [18], [19]. Low seizure threshold in this structure is likely due to a combination of intrinsic membrane properties and heavy intranuclear connections that give rise to regenerative positive feedback and synchronous discharge [16], [20], [21]. Behan and Haberly [16] suggested that this potentially epileptogenic mechanism may support information encoding by facilitating synaptic plasticity in EPN target areas and by providing rhythmic network activity patterns for an association of contextual with odor-related and other cue information.

Our findings may thus be of particular relevance for the modulation of emotional states through the amygdala. In fact, c-Fos mapping studies have identified the EPN as one of the brain areas that is most consistently activated by (cued and contextually) conditioned as well as unconditioned fear stimuli [22]–[24]. It is similarly activated by stimulation of the NPS-rich parabrachial nucleus [25], which relays nociceptive and visceral information to higher brain areas [26]. However, projections from the parabrachial nucleus do likely not provide NPS input to the EPN, as they target the central amygdala, but not its lateral and basolateral subnuclei or the EPN [27]. An alternative source of NPS for the EPN may be the medial amygdala [1], a structure that is also drastically activated by cued and contextually conditioned fear and is known to send substantial input into the EPN [28]. It should also be considered that the EPN may receive NPS innervation from the recently discovered group of NPS-positive cells in the locus coeruleus area [6]. Although it remains to be investigated whether the NPS expressing neurons in the locus coeruleus area indeed project to the EPN and whether or not they show similar functional properties as their noradrenergic counterparts, it is striking that increases of inhibition in the BLA recorded *in vivo* upon locus coeruleus stimulation could only partly be blocked by the beta-adreno receptor antagonist timolol [29]. The locus coeruleus is critically involved in the modulation of stress responses in the amygdala and their effect on memory formation [8]. The EPN on the other hand projects to brain regions that are critically involved in fear memory formation and retrieval, including all subdivisions of the hippocampus, the BLA [16], and the lateral entorhinal cortex. The most substantial projections from the amygdala to the EPN, in turn, originate in the lateral division of the amygdalo-hippocampal area [30]. It is striking, that in addition to the EPN, the lateral entorhinal cortex and amygdalo-hippocampal area are characterized by pronounced expression of NPSR [6]. Hence, the NPS system is well organized to integrate fear- and stress-related information from various sources and to modulate both direct and indirect information flow between the amygdala and hippocampus, via the EPN.

Following this line of evidence, we hypothesized a potential role of an NPS-activated EPN-to-BLA circuit *in vivo* for the retrieval and expression of fear memories. One of the best studied fear memory paradigms is classical fear conditioning, where animals very quickly learn to associate a previously neutral sensory stimulus or context with a coinciding aversive situation. In rodents, the so conditioned stimuli can elicit a behavioral response that comprises elements of freezing, risk-assessment and other defensive reactions according to the salience of the conditioned stimulus [31]. The circuitry and neural activity underlying different forms of such fear memory has been intensively studied. In general, it is considered that the amygdala is a critical site of information storage and plasticity during both cued and contextual conditioning, and that the hippocampus comes into play whenever complex, such as multimodal, contextual or temporal, information processing takes place. In addition, such functional distinction appears to exist within the amygdala itself, as auditory and nociceptive information converge on single neurons in the lateral subnucleus of the amygdala, while context information is relayed from the hippocampus mostly into the BLA [9]. Recent data suggest that amygdala and hippocampus interact reciprocally through a synchronization of network activities during both cued and contextual fear memory retrieval [32], [33]. The ability of the EPN to act as a rhythm generator may allow it to modulate or contribute to these network activities [16]. In fact, field oscillations (∼200 Hz) have been observed in the EPN and BLA in freely behaving rats, and it was suggested that this short-scale synchronous firing of subpopulations of projection neurons may organize the precise timing of multi-modal information flow in the BLA [34].

In our experiments, NPS administration to the EPN resulted in a selective disruption of contextual fear memory with reduction of both freezing and risk-assessment. At the same time, auditory cued fear memory remained largely unaffected by the treatment, excluding the possibility of a general disturbance of the retrieval or expression of conditioned fear. Moreover, NPS treated animals showed unaltered risk-assessment behavior during CS+ exposure and a normal response to neutral acoustic stimuli (CS−), which we determined as within-task measures of anxiety-like behavior. Together with an unaltered behavior in the elevated plus maze this rules out potentially confounding effects of changes in anxiety level during the retrieval session, as previously seen upon intracerebroventricular NPS injection at the dosage used [1]. In turn, these data demonstrate that the circuitry delineated in our study may not be critically involved in the modulation of general anxiety through NPS, but may rather selectively modulate fear memory functions mediated by amygdalo-hippocampal interactions. The behavioral change in our experiments is likely due to a local effect of NPS through the activation of NPS receptors in the EPN, but we cannot rule out a spreading to the NPSR-endowed lateral enthorhinal cortex. Further studies will be required to precisely delineate functionally distinct NPS circuits in the brain and their activation under different fear and anxiety conditions.

In conclusion, the present work describes a novel NPS sensitive pathway, controlling neural activity patterns in the BLA via the EPN. Endogenous NPS from the locus coeruleus area or medial amygdala similarly may enhance both excitatory and inhibitory drive onto BLA, resulting in firing of a subpopulation of projection neurons. A balancing of excitatory/inhibitory influences in the amygdala has previously been described for another neuropeptide, gastrin-releasing peptide (GRP) [35]. However, while GRP appears to be related to its persistence, NPS appears to control the retrieval and/or expression of contextual aspects in fear memory.

## Materials and Methods

### Slice preparation

All experiments were carried out in accordance with the European Committees Council Directive (86/609/EEC). Juvenile (P12–P22) GAD67-GFP (Δneo) mice [36] were anaesthetized with forene (isofluran, 1-Chloro-2,2,2-trifluoroethyl-difluoromethylether) and killed by decapitation. A block of tissue containing the amygdala was rapidly removed and placed in chilled oxygenated physiological saline containing (mM): KCl, 2.4; MgSO_4_, 10; CaCl_2_, 0.5; piperazine-N,N′-bis(ethanesulphonic acid) (PIPES), 20; glucose, 10; sucrose, 195 (pH 7.35). Coronal slices (250 µm thick) were prepared on a vibratome (Model 1000, The Vibratome Company, St. Louis, USA), and were incubated in standard artificial cerebrospinal fluid (ACFS) of the following composition (in mM): NaCl, 120; KCl, 2.5; NaH_2_PO_4_, 1.25; NaHCO_3_, 22; MgSO_4_, 2; CaCl_2_, 2; glucose, 10; bubbled with 95%O_2_/5% CO_2_.

### Recording techniques

Recordings were performed in the whole-cell mode on amygdala neurons as described previously [11]. Briefly, single slices including the BLA were transferred to a submerged chamber. Recordings were made using a patch-clamp amplifier (EPC-9, Heka, Lamprecht, Germany) under visual control of differential interference contrast infrared videomicroscopy (S/W-camera CF8/1, Kappa, Gleichen, Germany). A monochromator (Polychrome II, Till Photonics, Martinsried, Germany) connected to an epifluorescence system and a 40×/0.80 water immersion lens was used to identify interneurons by EGFP fluorescence. Projection neurons out of the BLA were identified by lack of fluorescence [36], as well as pyramidal-like morphology and spike frequency adaptation in response to prolonged depolarizations, as described previously [11]. Fluorescent interneurons occurring loosely scattered within the BLA showed typical electrophysiological properties as fast action-potentials (halfwidth of first spike: 1.2±0.1 ms, n = 13; halfwidth at the phase of spike broadening: 1.7±0.1 ms, n = 13) and sustained high firing frequencies (40.3±4.0 Hz, n = 13) as reported recently [10]. Intercalated neurons were identified by location along the medial border between the BLA and central amygdala as densely packed clusters of fluorescent cells [10]. Patch pipettes were pulled from borosilicate glass (GC150TF-10, Clark Electromedical Instruments, Pangbourne, UK) to resistances of 2–3 MΩ. A liquid junction potential of 10 mV of the pipette solution was corrected for. For recordings of spontaneous and miniature inhibitory postsynaptic currents (sIPSCs, mIPSCs), the pipette solution contained (in mM): Csgluconate, 117; CsCl, 13; MgCl_2_, 1; CaCl_2_, 0.07; EGTA, 1; HEPES, 10; MgATP, 3, NaGTP, 0.5 (pH 7.2 with KOH). For some experiments a pipette solution with high concentration of BAPTA was used, which contained (in mM): Csgluconate, 110; CsCl, 13; MgCl_2_, 1; CaCl_2_, 0.07; BAPTA, 10; HEPES, 10; MgATP, 3, NaGTP, 0.5 (pH 7.2 with KOH).

Application of the GABA_A_-antagonist (-)-bicuculline methiodide (10 µM) completely abolished sIPSCs/mIPSCs (n = 5), confirming mediation of IPSCs by GABA_A_ receptors and further ruling out contamination of recordings by glutamatergic events at times when the effects of NPS were tested (data not shown). EPSCs were recorded in the presence of (-)-bicuculline methiodide (10 µM). Spontaneous and miniature excitatory postsynaptic currents (sEPSCs, mEPSCs) and postsynaptic membrane currents were measured using an intracellular solution composed of (in mM): Kgluconate, 95; K_3_citrate, 20; NaCl, 10; HEPES, 10; MgCl_2_, 1; CaCl_2_, 0.1; EGTA, 1.1; MgATP, 3; NaGTP 0.5 (pH 7.2 with KOH). In the presence of 10 µM 6,7-Dinitroquinoxaline-2,3-dione (DNQX) in combination with 50 µM DL-2-Amino-5-phosphono-pentanoic-acid (AP5), EPSCs were blocked (projection neurons: n = 4; interneurons: n = 3), evidencing involvement of glutamatergic receptors. Miniature postsynaptic currents (mIPSCs, mEPSCs) and postsynaptic membrane currents were isolated in the presence of 1 µM tetrodotoxin (TTX). After obtaining the whole cell configuration, neurons were held routinely at −70 mV for EPSCs and mEPSCs or at 0 mV for IPSCs and mIPSCs, respectively.

A bipolar tungsten electrode (Science Products, Hofheim, Germany; WPI, Sarasota, FL) was placed on the surface of the slice above the external capsule or within the BLA for evoking EPSCs in interneurons and IPSCs in BLA projection neurons, respectively. EPSCs and IPSCs were elicited by two consecutive stimuli of 100 µsec duration delivered by a stimulus isolator (Isoflex, AMPI, Jerusalem, Israel), separated by an interstimulus interval of 50 msec. EPSCs were recorded in the presence of 10 µM bicuculline methiodide and in a modified ACSF with 3 mM Mg^2+^ and 1 mM Ca^2+^. IPSCs were recorded in the presence of AP-5 (50 µM) and NBQX (10 µM).

### Data analysis

Miniature postsynaptic currents were detected using the program ‘Mini-Analysis’ (Jaejin software, Leonia, NJ, USA). Cumulative histograms without bins were calculated within time periods of 30 s to 5 min duration containing at least 300 events before addition and whilst maximal effect of NPS. For determining changes in paired-pulse depression or facilitation, second IPSC or EPSC amplitudes were normalized according to first amplitudes (ratio peak 2/peak 1).

Statistical analysis was performed using nonparametric tests by Graph Pad Prism software (San Diego, CA, USA; Wilcoxon signed rank test for paired observations, Mann Whitney test for non paired observations). Data are presented as mean±SEM. Differences were considered statistically significant at p≤0.05.

### Drugs

NPS (200 nM) was applied only once to each slice for 2–3 min. Drugs were added to the external ACFS. For locally puffing NPS, patch pipettes were filled with 5 µM NPS dissolved in ACSF and connected to a picospritzer II (General Valve Corp., USA). Pipettes were advanced into the ventral EPN of coronal amygdala slices and NPS was locally puffed into the EPN with 5 psi for 10–20 s. Care was taken in order to avoid spillover of NPS into the BLA.

All substances were obtained from Sigma (Diesenhofen, Germany), except for NPS (Phoenix Europe GmbH, Karlsruhe, Germany), DNQX (Tocris, Bristol, UK), and TTX (Alomone, Jerusalem, Israel).

### Cut experiments

In some experiments, the BLA was mechanically dissected under a binocular microscope. The first type of cut separated the BLA ventrally from the medial and cortical amygdala, limited laterally by the region of endopiriform/piriform cortex. The second one was aligned along the external capsule detaching the capsule and the endopiriform cortex laterally (Figure 5). Isolated BLA slices were made by cutting off all tissue around the BLA.

### Analysis of conditioned fear behavior

To determine the behavioral significance of NPS-modulated BLA activity, we investigated the effect of NPS application to the EPN on the retrieval and expression of conditioned fear. All experiments were performed accordance with regulations through the German law (permission number 42502/2-618 UniMD).

**Figure 8:**
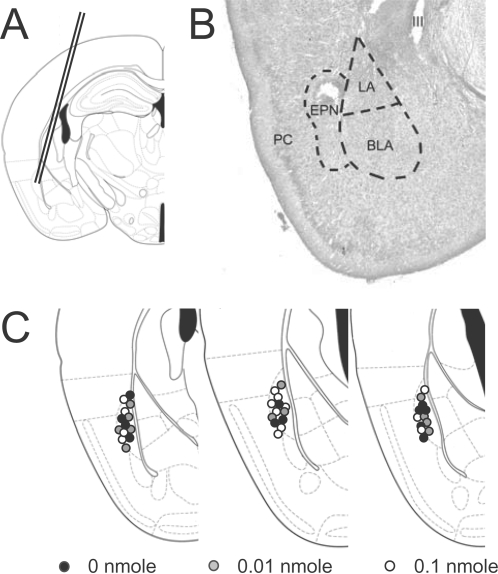
Probe location in the EPN. (A) Injection cannula were stereotactically implanted at a 10° angle, aiming at the dorsal portion of the EPN (B) Histological verification of probe location in a cresyl violet stained coronal section from a representative animal. A small lesion indicates the injection site, which is located in the dorsal EPN. LA, lateral amygdala; BLA, basolateral amygdala; EPN, endopiriform nucleus; PC, piriform cortex; III, third ventricle (C) Summary of injection sites in individual animals of all three test groups. Illustrations modified from [37].

Specificlally, ten-to-fourteen week old male C57BL/6BomTac mice (M&B Taconic, Berlin) were used. Animals were purchased at an age of six-seven weeks and kept in groups of 3–6 with a 12 h light/12 h dark cycle (lights on at 7:00 AM) with food and water ad libitum. One week before the experiment animals were separated and further on kept individually. All experiments were done during the dark phase of the cycle, with adaptations in the morning and afternoon and with training and retrieval session always between 1:00 PM and 5:00PM. On the first day of the experiment, mice were in two 6 min sessions accustomed to the training apparatus (TSE Bad Homburg, Germany) comprising a 36 cm×21 cm×21 cm light-blue acrylic glass arena with a grid floor for delivery of electric foot shocks. The arena was enclosed in an isolation cubicle containing a speaker, a ventilation fan providing fresh air, and background noise 70 dB sound-pressure level (SPL). During each adaptation session, a set of 6 acoustic stimuli (2.5 kHz sinus tone, 85 dB SPL, 10 s with 20 s inter-stimulus-intervals) was delivered to serve as auditory control stimulus (CS−). On the following day, after 2 min of habituation to the apparatus animals were fear conditioned with 3 conditioned stimuli (CS+; 10 kHz sinus tone, 85 dB SPL, 10 s with 20 s ISIs) that each co-terminated with an unconditional stimulus (US; 1 s scrambled foot shock, 0.4 mA). No CS− was presented during training. Three days after conditioning, guide cannula (length 10 mm diameter 1 mm) were implanted at 1.8 mm AP, 2.7 mm ML (10° angle) and 5.1 mm DV from Bregma under pentobarbital anesthesia (50 mg/kg) and fixed to the scull with dental cement. After recovery from surgery for one week, mice were injected with 0.1 nmole NPS (n = 14), 0.01 nmole NPS (n = 12) or vehicle (saline; n = 12) at a volume of 0.3 µl per animal. 15 min after injection, general anxiety level was assessed in an elevated plus maze (arm length 60 cm large, arm width 6 cm, wall height 15 cm, elevation 40 cm) in a single 5 min session under low-light conditions (100 Lux). Exploration of open and closed arms was evaluated using a video tracking software (ANY-maze, Stoelting, Wood Dale Il). 10 min later retrieval of conditioned fear memory was investigated. Animals were exposed to the training context for 2 min and then to sets of each four 10 s CS− and four 10 s CS+ with inter-stimulus-intervals of 20 s each. As measures of fear memory, freezing (complete immobilization except for respiratory movements) and risk-assessment behavior (overt watching, stretched attending) were evaluated off line using a time line version of the public domain program Wintrack (D. Wolfer, Univ. Zuerich). Treatment groups were compared with one-way analysis of variance (ANOVA) and post-hoc with Fischer's protected least significance difference (PLSD) test. After completion of experiments, cannula location was verified histologically; only correctly implanted animals were considered for statistical analysis (Fig. 8).
